# Phase 1 dose escalation of ONT-10, a therapeutic MUC1 vaccine, in patients with advanced cancer

**DOI:** 10.1186/2051-1426-1-S1-P240

**Published:** 2013-11-07

**Authors:** John Nemunaitis, Cynthia Bedell, Kevin Klucher, Alex Vo, Sam Whiting

**Affiliations:** 1 Mary Crowley Cancer Research Center, Dallas, TX, USA; 2 Oncothyreon, Inc., Seattle, WA, USA

## Background

Mucin 1 (MUC1), a glycoprotein normally expressed at low levels on the apical borders of secretory epithelial cells, is overexpressed and aberrantly glycosylated in many cancers. ONT 10 is a therapeutic peptide vaccine incorporating a synthetic glycolipopeptide MUC1 antigen, M40Tn6, and novel synthetic TLR-4 agonist, PET Lipid A, in a liposomal formulation designed to elicit antibody and cellular immune responses.

## Methods

A phase 1 study was initiated to evaluate the safety and tolerability of ONT-10, as well as cellular and humoral immune responses and antitumor activity. Patients (pts) with incurable solid tumors associated with MUC1 expression were eligible. Cyclophosphamide 250 mg/m2 IV was given on day -3 followed by ONT-10 at the cohort-specific dose (250 µg, 500 µg or 1000 µg) subcutaneously day 1 and then Q2W for 4 total doses or QW for 8 total doses in a 3+3 dose escalation design. Immune response was assessed by serum titers of MUC1-specific antibodies using M40Tn6 ELISA and by MUC1-specific ELISPOT for interferon gamma. Tumor response was assessed by RECIST 1.1 and immune-related response criteria (irRC). Pts without progressive disease by irRC were eligible for a maintenance protocol to receive ONT-10 every 6 weeks.

## Results

The study is ongoing and 28 pts have been treated. Diagnoses were: ovarian/primary peritoneal (n=10), pancreatic (n=5), colorectal (n=3), endometrial (n=3), breast (n=2), lung, bladder, cervical, duodenal, and prostate (n=1 each). Median prior lines of therapy 4 (range 1 - 11); median age 61.5 years (range 35 - 77); all pts had ECOG status of 0/1. No DLTs have occurred at doses up to 1000 µg Q2W and 500 μg QW. 90% of AEs have been Grade 1-2; the most common (≥20%) being fatigue (40%), abdominal pain (28%), nausea (28%) and constipation (24%). The most common treatment-related AEs (TRAEs) have been fatigue (32%) and injection site reactions (20%) and all TRAEs have been Grade 1-2. MUC1 specific antibody responses were seen in the majority of pts. Cellular response assessment is ongoing. Best tumor response in 25 evaluable pts was SD (17 pts; 68%) and PD (8 pts; 32%). One pt with ovarian cancer had 16% tumor shrinkage. SD ≥ 6 mo was seen in 28% of patients including ovarian/primary peritoneal (n=3) and endometrial, breast, colon, pancreatic (n=1 each). 17 pts have enrolled on the maintenance study.

## Conclusions

In a diverse population of late stage cancer pts, ONT-10 was well tolerated at up to 1000 µg Q2W and 500 µg QW. Prolonged disease control and encouraging immune responses have been seen. Updated results and immune data will be presented.

